# Targeting Gut Microbiome Dysbiosis as a Potentially Effective Therapeutic Approach for the Treatment of Heart Failure

**DOI:** 10.31083/RCM47146

**Published:** 2025-11-27

**Authors:** Morris Karmazyn

**Affiliations:** ^1^Department of Physiology and Pharmacology, University of Western Ontario, London, ON N6G 2X6, Canada

## 1. Introduction: The Gut Microbiome and Dysbiosis

Gastrointestinal health is largely regulated by the nature of the bacterial and viral content inhabiting the intestinal tract, generally referred to as the gut microbiome [1]. The nature of the gut microbiome can markedly affect the disease process and an imbalance in these microorganisms, referred to as dysbiosis, can contribute to various diseases. Dysbiosis can occur as a consequence of numerous factors such as genetics, dietary and lifestyle habits, diseases, pharmacological agents such as antibiotics, as well as others [2]. Dysbiosis can negatively affect the function of numerous distal organs including the heart *via* a process generally referred to as the gut-heart axis. This editorial discusses the gut-heart axis and how dysbiosis can affect heart failure. Potential therapeutic approaches towards mitigating heart failure *via* manipulation of the gut microbiome and dysbiosis are reviewed.

## 2. General Mechanisms Underlying the Gut-Heart Axis

The heart can be affected by gut dysbiosis. There is currently extensive evidence that heart disease can be markedly affected by disruption of the gut microflora. Therefore, modulation of the gut microbiome can represent an attractive and effective therapeutic approach for the treatment of various cardiovascular pathologies. Among these is heart failure, which occurs *via* enhanced myocardial remodelling and hypertrophy which can be augmented by the gut-heart axis [3, 4, 5].

**Fig. 1.**
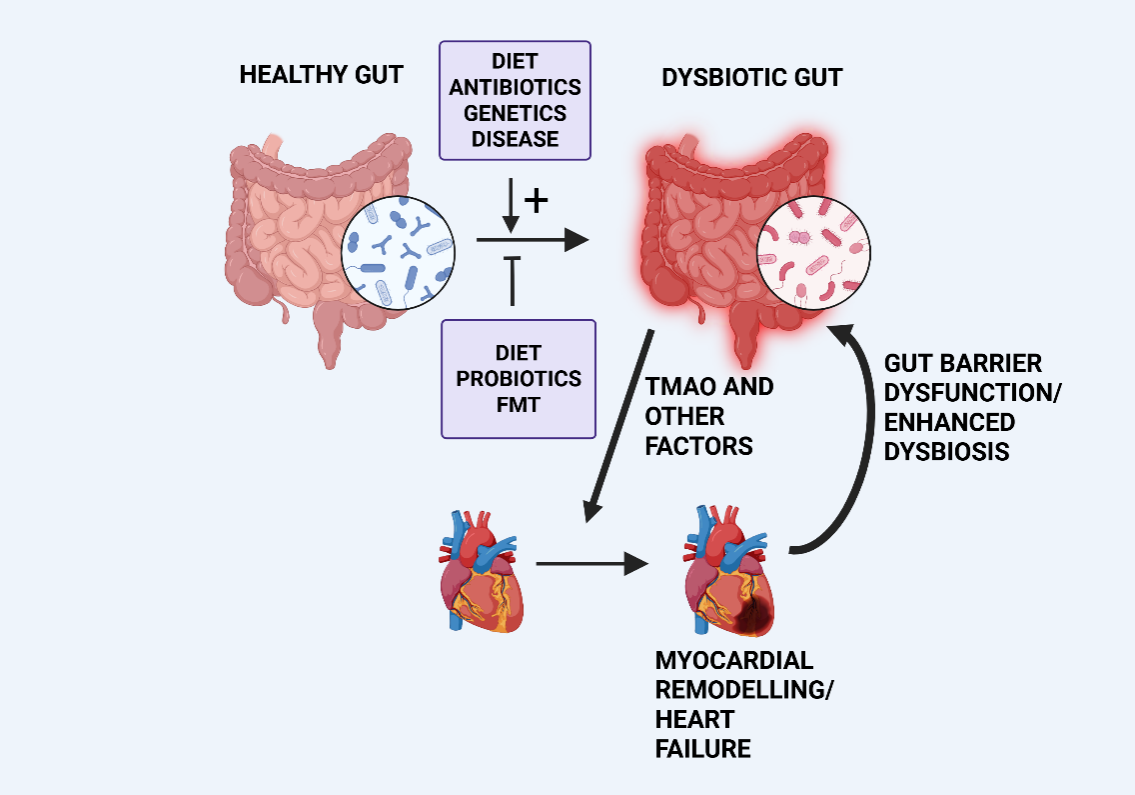
**Simplified diagram illustrating dysbiosis-induced cardiac remodelling and the reciprocal nature of the gut-heart axis**. Various factors, indicated in the upper box, can promote gut dysbiosis resulting in the release of factors promoting myocardial remodelling and heart failure, as discussed in the text. The remodelled heart can further promote dysbiosis *via* various mechanisms, principally *via* gut barrier dysfunction. Potential reduction of dysbiosis can result from dietary intervention, administration of probiotics or by Fecal Microbiota Transplantation (FMT) as indicated in the lower box. TMAO, trimethylamine N-oxide. Created with BioRender.com.

The mechanisms underlying the gut-heart axis have been well studied and with substantial evidence documented in both animal as well as clinical studies [3, 4, 5, 6]. Altering the gut microbiota to a more favourable microorganism profile results in improved cardiovascular status. Dysbiosis is associated with enhanced myocardial oxidative stress, proinflammatory responses, as well as alterations in the myocardial epigenetics profile, all of which contribute to the onset of heart failure [7, 8, 9, 10]. These negative effects can occur *via* both direct and indirect mechanisms. Dysbiosis is associated with intestinal dysfunction including enhanced inflammation and intestinal wall permeability resulting in the release of pathogenic toxins into the bloodstream which directly exert cardiotoxic effects as has been shown clinically in heart failure patients [11, 12]. The second major mechanism for cardiac impairment associated with dysbiosis involves the production of microorganism-derived toxic metabolites released into the circulation. One of the most widely studied of these metabolites is trimethylamine N-oxide (TMAO), a dietary choline-derived metabolite which produces a plethora of effects including increased cardiac fibrosis partially via activation of the NLRP3 inflammasome, increased inflammation, endothelial dysfunction, and progression of atherosclerosis [13] as well as directly promoting cardiac hypertrophy [14]. The latter effects appear to be dependent on multifaceted mechanisms including elevations in intracellular calcium concentrations and TGF-β1/Smad3 signalling [15]. TMAO is elevated in heart failure patients [16]. Choline or a TMAO-supplemented diet has been shown to enhance heart failure in a mouse pressure overload model produced by thoracic aorta banding [17]. As has been demonstrated in a number of experimental heart failure models [18, 19], it is important to emphasize that the relationship between dysbiosis and heart failure is reciprocal in that heart failure likely induces or enhances dysbiosis and thus can further contribute to cardiac dysfunction and may be a key mechanism contributing to the increased incidence of inflammation seen in heart failure patients [20]. Many studies have now shown that heart failure alters the gut microbiota [20] predominantly *via* changes in cardiovascular hemodynamics resulting in gut ischemia. The resultant gut barrier dysfunction leads to further release of gut-derived pro-remodelling factors into the circulation (reviewed in [21]). A summary of the gut-heart axis is illustrated in Fig. 1.

## 3. Therapeutic Potential and Approaches Aimed at Modifying Dysbiosis and the Gut-Heart Axis for the Treatment of Heart Failure 

The phenomenon of dysbiosis affecting cardiac function and contributing to heart failure has led to a number of potential therapeutic interventions which could result in effective adjunctive treatments for heart disease including heart failure. One such approach is the administration of probiotics which would enhance the composition of the gut microbiota with so-called beneficial microorganisms thus attenuating the heart failure process. Such a benefit has been demonstrated in a number of experimental animal models including rats subjected to 30 minutes of ischemia followed by 2 hours of reperfusion in which administering the probiotic supplement Goodbelly® (containing *L. plantarum* and *Bifidobacterium lactis*) for 14 days prior to surgery significantly reduced infarct size in these animals [22]. The author’s laboratory demonstrated that the administration of the probiotic *Lactobacillus rhamnosus* GR-1 reduced myocardial hypertrophy and improved left ventricular function in rats subjected to 6 weeks of coronary artery ligation [23]. Administering a synbiotic (combination of probiotic and prebiotic) attenuated myocardial structural changes and hypertrophy in a porcine model of the metabolic syndrome [24]. In a small clinical study of heart failure patients (NYHA Class II and III), administration of the probiotic *Saccharomyces boulardii* resulted in an improvement in various parameters including a significant increase in left ventricular ejection fraction [25], although a recent clinical evaluation of this probiotic in 46 heart failure patients revealed no improvement following three-month of treatment [26]. Recently, a multistrain probiotic was shown to reduce sarcopenia and improve physical capacity in heart failure patients [27]. The composition of this multistrain probiotic can be found in reference [27].

Fecal microbiota transplantation offers another approach towards improving the gut microbiome and potentially mitigating myocardial remodelling. Fecal transplantation, first recorded hundreds of years ago, has been shown to favourably influence various diseases [28] and was demonstrated to reduce the severity of heart failure in a number of experimental animal models [29, 30].

## 4. Conclusions and Future Directions

Substantial evidence suggests that modifying the gut microbiome by reducing dysbiosis contributes not only to gastrointestinal health but also to ameliorating various pathologies including cardiovascular diseases such as heart failure. Interest in the gut-heart axis, particularly within the past ten years, has been impressive based on the number of publications cited in PubMed. Experimental animal studies strongly support the concept of the gut-heart axis and its contribution to cardiac pathology due to dysbiosis. This is derived to a large degree from cardiac benefits seen through the administration of probiotics as well as improvement in cardiac parameters in experimental heart failure following fecal transplantation. Application of this concept to the clinical scenario is important particularly in view of the limited data currently available from studies derived from small patient populations. Thus, clinical evaluation of probiotics in heart failure patients in large scale clinical studies is warranted. Addition of probiotics to standard heart failure therapy or the use of other approaches to reduce gut dysbiosis would be a reasonable initial approach in order to ascertain clinical efficacy for the treatment of heart failure particularly in view of the discordant clinical results, as noted in the previous section. Nevertheless, clinical evaluation presents complex challenges when compared to animal studies, with factors such as co-morbidities, patient demographics and many other factors coming into play. Yet these clinical trials, particularly with appropriate patient recruitment, are critical to clearly assess the efficacy of reducing dysbiosis for the treatment of heart failure.
